# Cross-border collaboration, communication, and research frontiers on biologics in chronic rhinosinusitis from 2004 to 2023

**DOI:** 10.3389/fdata.2024.1428074

**Published:** 2024-12-02

**Authors:** Guan-Jiang Huang, Zhi-Jun Fan, Biao-Qing Lu

**Affiliations:** Department of Otorhinolaryngology Head and Neck Surgery, Zhongshan Hospital of Traditional Chinese Medicine, Affiliated to Guangzhou University of Chinese Medicine, Zhongshan, Guangdong, China

**Keywords:** chronic rhinosinusitis, biologics, bibliometric analysis, collaboration, frontier

## Abstract

**Objective:**

Biologics are considered as a promising novel treatment option for patients with chronic rhinosinusitis who failed with the standard of care (medical therapy and surgical interventions). This bibliometric analysis was performed to explore cross-border collaboration, communication, and research frontiers on biologics in chronic rhinosinusitis.

**Methods:**

Original research publications on biologics in chronic rhinosinusitis were retrieved from the Science Citation Index-Expanded (SCI-E) database in the Web of Science Core Collection between 2004 and 2023. Using CiteSpace and R software, the country/region, author, institution, journal, reference, and keywords were extracted to analyze the research focus and global trends in this field.

**Results:**

Research articles exhibited a consistent rising trend from 2004 to 2023, especially the period between 2020 and 2023. Most articles were published by authors from the USA. The USA was the most cited country, enjoying the most active cooperation with other countries/regions. Bachert C owned the most publications and collaborations. Ghent University and Karolinska Institute had the most collaborations with other institutions. *Journal of Allergy and Clinical Immunology* and *Allergy* published the most articles and were the most co-cited journals. Research frontiers on biologics in chronic rhinosinusitis would focus on efficacy, quality of life, safety, children, management, etc.

**Conclusions:**

This bibliometric analysis displayed the overall situation and global trend on biologics in chronic rhinosinusitis. The visualization analysis of publications could assist researchers rapidly in understanding the hotspots and trends. Further research is warranted to determine the long-term effects and side effects of biologics in chronic rhinosinusitis.

## 1 Introduction

Chronic rhinosinusitis affected about 4.3%–12.5% of the population worldwide (Hopkins, 2019; Agache et al., 2021; Kato et al., 2022). Patients with chronic rhinosinusitis often require long-term use of systemic corticosteroid and/or repeated sinus surgery, suffering a high symptom burden, a huge economic burden, and a poor quality of life (Bachert et al., 2019; Chong et al., 2020; Agache et al., 2021). The management of these patients remains challenging. The successful application of biologics in asthma promoted the use of similar biologic treatments for chronic rhinosinusitis (Gevaert et al., 2013; Iqbal et al., 2020; Mullol et al., 2022).

Nowadays, biologics are considered as a promising novel treatment option for patients with chronic rhinosinusitis who fail with the standard of care (medical therapy and surgical interventions) (Gevaert et al., 2013; Mullol et al., 2022). A recent network meta-analysis proved that biologics and endoscopic sinus surgery both could significantly improve key nasal outcomes in chronic rhinosinusitis with nasal polyps (Chen et al., 2023). More particularly, dupilumab obtained better efficacy than endoscopic sinus surgery on SNOT-22 scores at 1 year. A systematic review summarized that treatment with omalizumab and mepolizumab improved endoscopic nasal polyp score and symptoms score in patients compared with placebo (Iqbal et al., 2020). Nasal polyp size was reduced after using reslizumab, especially in patients with raised intranasal interleukin-5 (IL-5) levels. Dupilumab reduced 70% of endoscopic nasal polyp score. These biologics targeted various inflammatory markers that were involved in the pathophysiology of chronic rhinosinusitis, bringing considerable clinical improvement. Therefore, a general overview of developments of biologics in chronic rhinosinusitis is required.

In the current era filled with the overwhelming abundance of information, it is increasingly arduous to maintain a comprehensive understanding of the field. As a pioneering method, the bibliometric analysis would be an excellent option to evaluate quantitatively the impact of research publications on the selected research field in terms of countries/regions, collaboration, journals, institutions, authors, and keywords (Oelrich et al., 2007; Jiang et al., 2023). Bibliometric analysis has been gradually applied in various research domains (Bornmann and Leydesdorff, 2014). Compared with the traditional systematic review and the meta-analysis, bibliometric analysis can more systematically and visually reveal the current trend and development of research topics (Bould et al., 2014; Jiang et al., 2023). Furthermore, Bibliometric analysis can identify highlights and “hotspots” to generate perspectives to guide future research orientations for potential researchers in this field.

To provide an up-to-date global overview regarding cross-border collaboration, communication, and research frontiers on biologics in chronic rhinosinusitis, we performed this bibliometric analysis based on research publications from 2004 to 2023.

## 2 Materials and methods

### 2.1 Data sources and search strategies

Owing to the capability to generate high-quality academic publications, the Web of Science Core Collection (WoSCC, https://www.webofscience.com/wos/) is usually utilized to conduct the bibliometric analysis (Bould et al., 2014). The search terms contained “chronic rhinosinusitis,” “nasal polyp,” “anti-IgE monoclonal antibody,” etc. The Science Citation Index-Expanded (SCI-E) database in the WoSCC database was extensively searched for relevant data from 2004 to 2023. Original research articles were solely included. Non-English articles and other types of publications were excluded. Two authors separately analyzed the data. Conflicts were settled with the assistance of the third author. We focused on information about titles, authors, countries, journals, institutions, references, etc.

### 2.2 Bibliometric analysis and visualization

Before being loaded into the analysis software, WoSCC data had been converted to the corresponding format. CiteSpace software (version 5.7.R1, 64-bit, Drexel University, Philadelphia, PA, USA) and “bibliometrix” package (https://www.bibliometrix.org/home/) based on R software (version 4.3.2, https://www.r-project.org, The R Foundation) were used to analysis articles, countries, institutions, authors, journals, keywords, etc., as well as to visual the results. The visualizations were drawn for displaying the results.

## 3 Results

### 3.1 Searching and selection procedure

**Figure 1:**
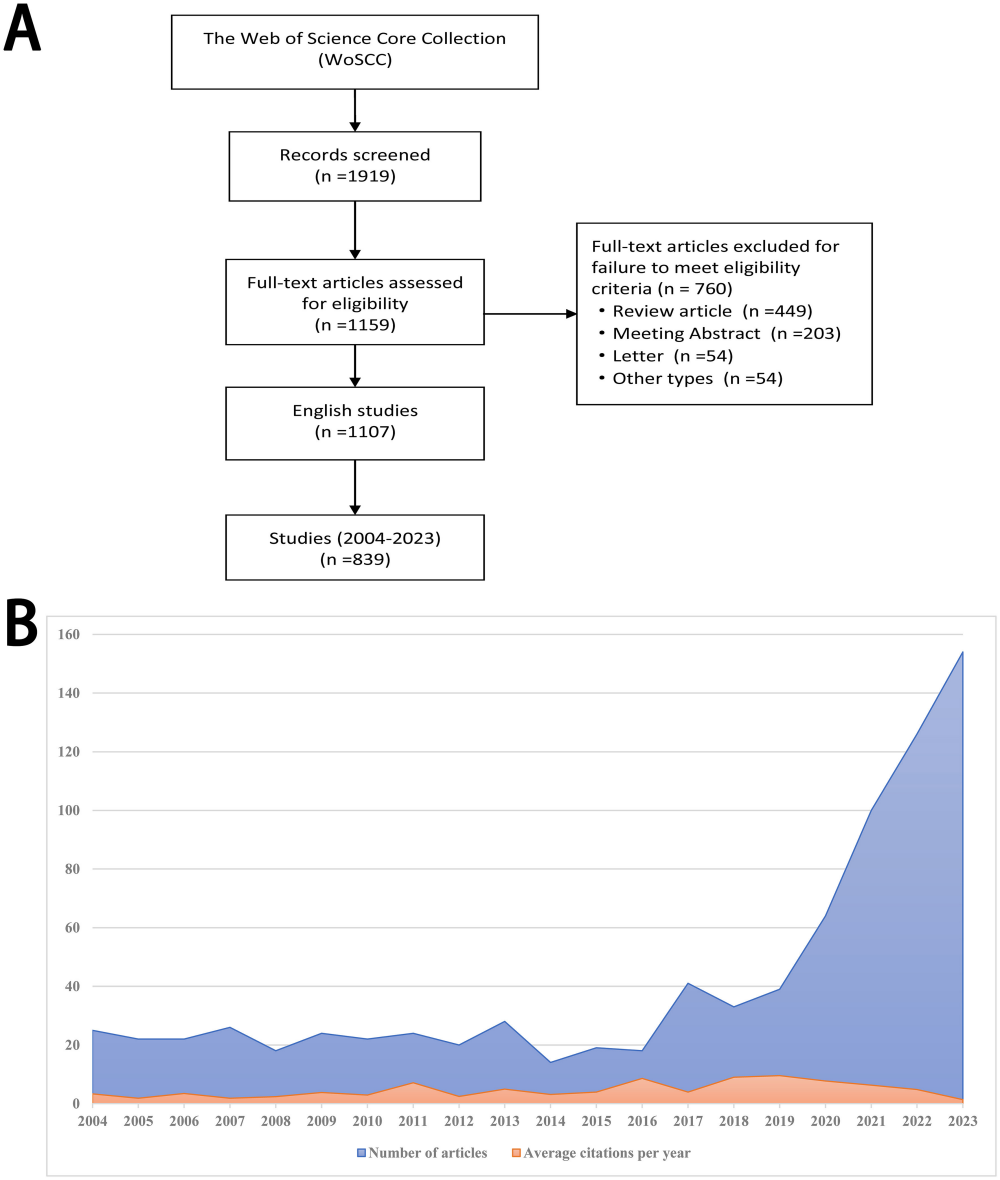
Flow diagram and annual production. **(A)** Flow diagram of publication searching and selection procedure. **(B)** The annual production of research publications linked to biologics in chronic rhinosinusitis from 2004 to 2023.

As was shown in Figure 1A, 1,919 related publications were screened from the WoSCC database. After excluding review articles, meeting abstracts, letters, and other types, 1,159 research articles met eligibility criteria. Among them, 839 English studies published between 2004 and 2023 were finally included.

### 3.2 Annual volume and growth tendency

Research articles linked to biologics in chronic rhinosinusitis exhibited a consistent rising trend from 2004 to 2023, especially the period between 2020 and 2023 (Figure 1B). Average citations per year also showed an overall rising trend (Figure 1B).

### 3.3 Distribution and cooperation of research countries/regions

**Figure 2:**
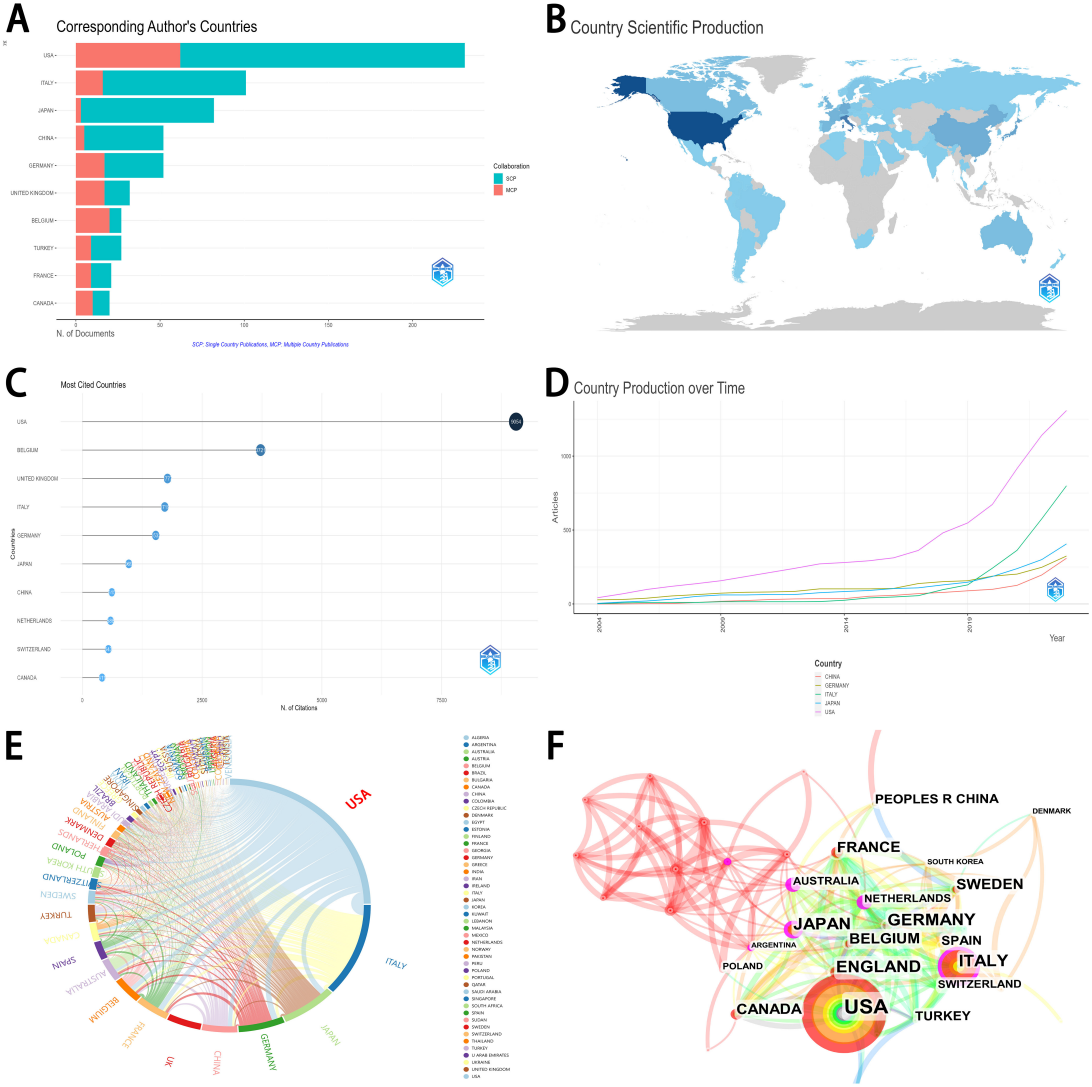
Distribution and cooperation of research countries/regions. **(A)** The distribution of top 10 research countries/regions. **(B)** The distribution visualization of research countries/regions in the world map. The depth of color represents the level of production in a country. The country with the deeper color would acquire higher production. **(C)** The number of citations in the top 10 research countries/regions. **(D)** The trend on country production over time in the top 5 research countries/regions. **(E)** Chord maps for co-operation of research countries. Each outer curve represents a country. Line thickness directly correlates to the strength of collaboration among various countries. **(F)** The co-operation of research countries/regions. The size of each sphere represents the number of publications from that country. SCP, single country publications; MCP, multiple country publications.

Table 1 and Figure 2 displayed the distribution and cooperation of the top 10 research countries/regions. Most articles were published by authors from the USA (231, 36.96%), followed by Italy (101, 16.16%), Japan (82, 13.12%), China (52, 8.32%), and Germany (52, 8.32%). Figure 2A depicted the distribution of single country publications (SCP) and multiple country publications (MCP) in the top 10 research countries/regions. Figure 2B showed the visual map of country scientific production. Among the top 10 countries, Belgium obtained the highest MCP ratio (0.74). The most cited countries were the USA (9,054 citations), followed by Belgium (3,721 citations), the United Kingdom (1,777 citations), Italy (1,719 citations), Germany (1,532 citations), etc. (Figure 2C). The top five productive countries all acquired a consistent rising trend over time (Figure 2D). USA, Italy, and Japan performed the most cooperation in research with other countries (Figures 2E, F).

**Table 1:** Distribution and cooperation of top 10 research countries/regions contributing to publications.

**Country**	**Articles**	**% (in the top 10 research countries/regions)**	**SCP**	**MCP**	**MCP ratio**
USA	231	35.81%	169	62	0.268
Italy	101	15.66%	85	16	0.158
Japan	82	12.71%	79	3	0.037
China	52	8.06%	47	5	0.096
Germany	52	8.06%	35	17	0.327
United Kingdom	32	4.96%	15	17	0.531
Belgium	27	4.19%	7	20	0.741
Turkey	27	4.19%	18	9	0.333
France	21	3.26%	12	9	0.429
Canada	20	3.10%	10	10	0.500

SCP, single country publications; MCP, multiple country publications.

### 3.4 Distribution and cooperation of authors

**Figure 3:**
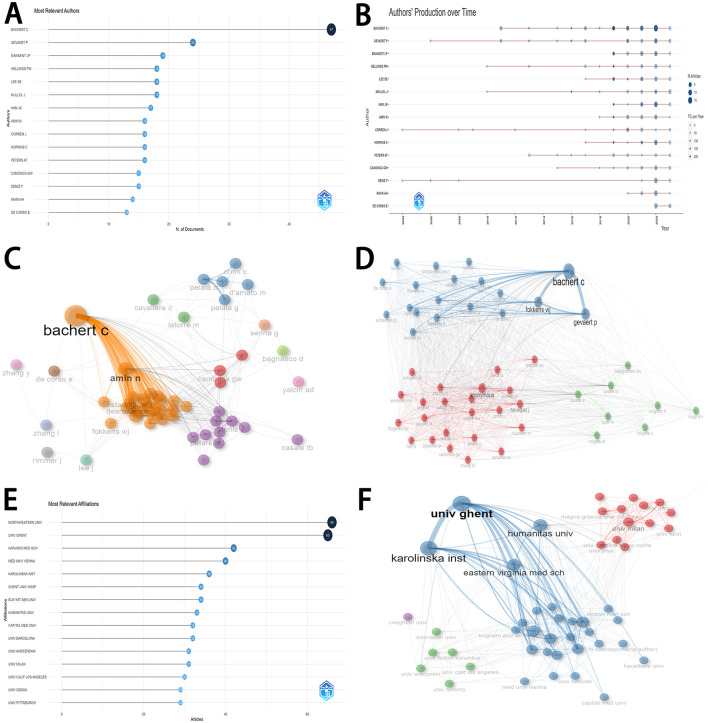
Distribution and cooperation of authors and institutions. **(A)** The distribution of the top 15 authors. **(B)** The detailed distribution of authors' production over time in the top 15 research countries/regions. **(C)** The network of collaboration among authors. **(D)** The network of co-cited authors. **(E)** The distribution of top 15 research institutions. **(F)** The network of collaboration among institutions.

Figure 3A displayed the production of the top 15 authors. Most articles were published by Bachert C (47 articles), followed by Gevaert P (24 articles), and Mannent LP (19 articles). Among the top 15 authors, Bachert C obtained a consistently rising production over time (Figure 3B). Figure 3C depicted Bachert C and Amin N played vital roles in the network of collaboration between authors. Bachert C, Gevaert P, and Fokkens WJ were considered as the most important roles in the network of co-cited authors (Figure 3D).

### 3.5 Distribution and cooperation of institutions

Figure 3E displayed the production of the top 15 institutions. Most articles were published by Northwestern University (66 articles), followed by Ghent University (65 articles), Harvard Medical School (42 articles), Medical University of Vienna (40 articles), and Karolinska Institute (36 articles). Among these institutions, Ghent University and Karolinska Institute played vital roles in the network of collaboration (Figure 3F).

### 3.6 Analyses of journals and references

**Figure 4:**
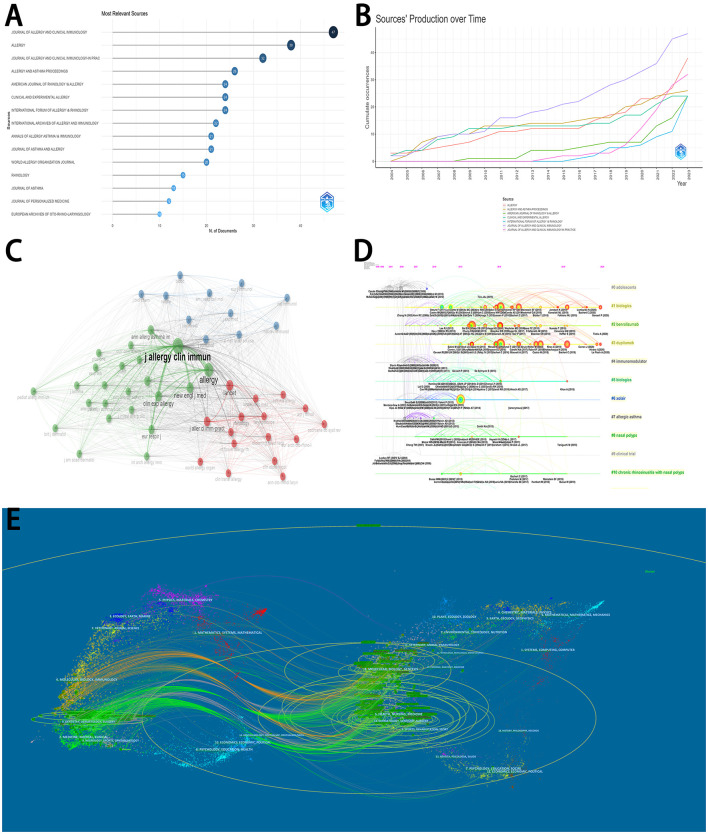
Analyses of journals and references. **(A)** The distribution of top 15 journals. **(B)** The detailed distribution of the production over time in the top 7 journals. **(C)** The network of co-cited journals. **(D)** The timeline view of the co-cited clustering plot with cluster labels on references. **(E)** The dual-map overlay of journals on biologics for chronic rhinosinusitis.

Figure 4A displayed the production of the top 15 journals. Most articles were published in *Journal of Allergy and Clinical Immunology* (47 articles), followed by *Allergy* (38 articles), *Journal of Allergy and Clinical Immunology-in Practice* (32 articles), and *Allergy And Asthma Proceedings* (26 articles). The top seven productive journals all published related articles with a consistent rising trend over time (Figure 4B). Among all journals, *Journal of Allergy and Clinical Immunology* and *Allergy* played vital roles in the network of co-cited journals (Figure 4C).

The timeline view of the clustering plot may indicate emerging research hotspots on biologics for chronic rhinosinusitis (Figure 4D). The application of Benralizumab, dupilumab, and Xolair would be current hotspots to treat chronic rhinosinusitis. The top 15 cited articles were listed in Table 2. The study by Bachert et al. (2019) had the most citations in *Lancet* (676 citations), followed by Bachert et al. (2016) in *JAMA* (548 citations), and Castro et al. (2011) in *American Journal of Respiratory and Critical Care Medicine* (533 citations).

**Table 2:** Top 15 cited articles.

**Articles**	**Journal**	**DOI**	**Total citations**	**Total citations per year**
Bachert C, 2019	Lancet	10.1016/S0140-6736(19)31881-1	676	135.20
Bachert C, 2016	JAMA	10.1001/jama.2015.19330	548	68.50
Castro M, 2011	American Journal of Respiratory and Critical Care Medicine	10.1164/rccm.201103-0396OC	533	41.00
Gevaert P, 2013	Journal of Allergy and Clinical Immunology	10.1016/j.jaci.2012.07.047	494	44.91
Akdis CA, 2013	Journal of Allergy and Clinical Immunology	10.1016/j.jaci.2013.02.036	415	37.73
Hanania NA, 2011	Annals of Internal Medicine	10.7326/0003-4819-154-9-201105030-00002	415	31.92
Gevaert P, 2011	Journal of Allergy and Clinical Immunology	10.1016/j.jaci.2011.07.056	402	30.92
Beck LA, 2004	Journal of Allergy and Clinical Immunology	10.1016/j.jaci.2004.06.032	375	18.75
Gevaert P, 2006	Journal of Allergy and Clinical Immunology	10.1016/j.jaci.2006.05.031	337	18.72
Bal SM, 2016	Nature Immunology	10.1038/ni.3444	335	41.88
Bachert C, 2017	Journal of Allergy and Clinical Immunology	10.1016/j.jaci.2017.05.044	328	46.86
Gevaert P, 2020	Journal of Allergy and Clinical Immunology	10.1016/j.jaci.2020.05.032	324	81.00
Detke HC, 2018	Neurology	10.1212/WNL.0000000000006640	306	51.00
Vignola AM, 2004	Allergy	10.1111/j.1398-9995.2004.00550.x	305	15.25
Ordovas-Montanes J, 2018	Nature	10.1038/s41586-018-0449-8	277	46.17

With citing journals on the left and cited journals on the right, the dual-map overlay of journals displayed the distribution of journals' links (Figure 4E). The colored routes can indicate the stated relationships. Due to the limited number of available articles, the trend of this dual-map overlay needed to be further explored.

### 3.7 Analysis of keywords

**Figure 5:**
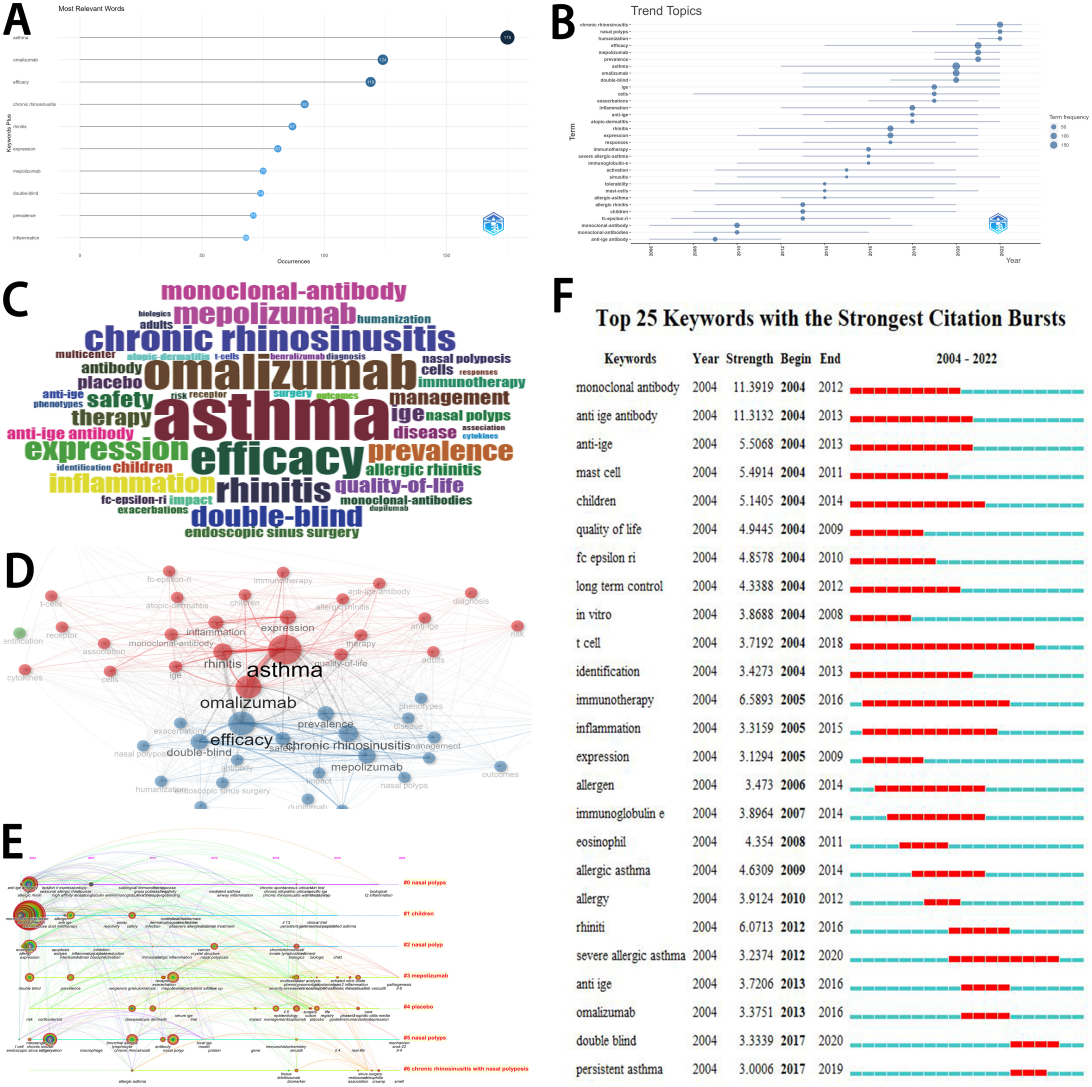
Analyses of keywords. **(A)** The occurrences of top 10 keywords. **(B)** The trend topics. **(C)** The visualization of the importance of various keywords. **(D)** The network of keyword co-occurrence. **(E)** The timeline view of the co-cited clustering plot with cluster labels on references. The size of the sphere is determined by keyword frequency. This visualization may offer insights into the number of keywords within each cluster. **(F)** Keywords with the strongest citation bursts. The chronology is shown by a blue line that cuts through 1 year. The burst period is displayed by a red reflection line marking the start year and finish year.

Figure 5A displayed the occurrences of the top 10 keywords. Most articles were focused on asthma (175 articles), followed by omalizumab (124 articles), efficacy (119 articles), chronic rhinosinusitis (92 articles), and rhinitis (87 articles). The trend topics were showed in Figure 5B. Figure 5C visually displayed the importance of various keywords. Figure 5D showed the network of keyword co-occurrence. Figure 5E dispalyed the timeline view of the co-cited clustering plot with cluster labels on references. Keyword bursts were detected in Figure 5F. Between 2004 and 2012, “monoclonal antibody” had the highest burst strength (11.39). Between 2013 and 2016, “omalizumab” had the highest burst strength (3.38). Between 2017 and 2020, “double-blind” had the highest burst strength (3.33).

## 4 Discussion

After searching the currently available publications, this was the first application of qualitative bibliometric methods of biologics in chronic rhinosinusitis. In this systematic bibliometric analysis, we analyzed annual volume and growth tendency, research countries/regions, authors, institutions, journals, references, and keywords.

Research articles linked to biologics in chronic rhinosinusitis exhibited a dramatic rising trend from 2020 to 2023. The USA contributed >35% of research articles. Moreover, the USA was the most cited country, enjoying the most active cooperation with other countries/regions. Nevertheless, many publications were contributed by Italy, Japan, and China with fewer collaborations. It indicated that these similar countries should enhance international cooperation with other countries, which would increase more high-quality research articles in this field.

Being far ahead of other authors, Bachert C owned the most publications and collaborations in the field linked to biologics in chronic rhinosinusitis. Most articles of the top 15 authors were published between 2019–2023. This aspect indicated that biologics in chronic rhinosinusitis were paid more and more attention in the treatment of chronic rhinosinusitis in recent years. Bachert C, Gavaert P, and Fokkens WJ were the top three co-cited authors.

In terms of the top five institutions, Northwestern University and Harvard Medical School originated from the USA, while Ghent University, Medical University of Vienna, and Karolinska Institute originated from the Netherlands, Austria, and Sweden, respectively. This aspect reflects an globally urgent demand of biologics in the treatment chronic rhinosinusitis. Ghent University and Karolinska Institute had the most collaborations with other institutions, which provided a worthwhile lesson for other institutions.

The most relevant journals linked to biologics in chronic rhinosinusitis were *Journal of Allergy and Clinical Immunology, Allergy*, and *Journal of Allergy and Clinical Immunology-In practice*. Through these high-quality journals, more and more researchers are paying attention to biologics in chronic rhinosinusitis. *Journal of Allergy and Clinical Immunology* and *Allergy* were also the most co-cited journals. These journals led the related research in the field. The top 15 co-cited references demonstrated that the clinical application of biologics in chronic rhinosinusitis was eager to be validate the efficacy and the quality of life. The top 10 high-frequency keywords demonstrated that the researchers focused on the clinical efficacy of various biologics in chronic rhinosinusitis, with various double-blind trials. Based on the gratifying achievements of basic experimental research, the researchers were gradually shifting toward clinical practice, aiming to guide the use of various biologics in suitable patients.

Monoclonal antibodies could be a promising therapy for patients with corresponding phenotypes and endotypes (Avdeeva and Fokkens, 2018; Chapurin et al., 2022). Biologics (omalizumab, dupilumab, benralizumab, etc.) have become an emerging application for chronic rhinosinusitis in recent years (Castro et al., 2011; Bachert et al., 2016, 2019). Current evidence proved that biologics can reduce the size of nasal polyps and improve patients' quality of life (Chong et al., 2020; Fokkens et al., 2023). The application of biologics can decrease the use of oral glucocorticoids and the need for reoperation (Bachert et al., 2021; Thamboo et al., 2021). Furthermore, patients' quality of life was also concerned in the biologics era (Mullol et al., 2022). Significantly impaired quality of life has been used as a criterion for the patients' indication to use biologics. Current biologics are often applied to patients with intractable chronic rhinosinusitis with nasal polyps. Murdaca et al. pointed out the importance of pharmacogenomics in the choice of tailored biologics as TNF-alpha inhibitors in psoriatic arthritis (Murdaca et al., 2014a,b). Thus, it is urgent to explore novel therapeutic modalities (Fokkens et al., 2023). This analysis indicated that research frontiers on biologics in chronic rhinosinusitis would focus on efficacy, quality of life, safety, children, management, etc.

Unlike previous studies (meta-analysis or narrative reviews), the bibliometric analysis provides a more intuitive visualization of evidence displaying research focal points and global trends across diverse dimensions. Meanwhile, third limitations should be also noticed. Firstly, our results were analyzed based on the currently available publications in the database. Continuous updating of publications might lead to different results. Secondly, the omission of other publication types (books, chapters, letters, etc.) and non-English articles may result in some biases in this analysis. Thirdly, due to the large volume of publications, it was not feasible to comprehensively discuss each publication, potentially affecting the depth of the subarea analysis.

## 5 Conclusions

In summary, this bibliometric analysis displayed the overall situation and global trend on biologics in chronic rhinosinusitis. The visualization analysis of publications could assist researchers rapidly in understanding the hotspots and trends. Further research is warranted to determine the long-term effects and side effects of biologics in chronic rhinosinusitis.

## Data Availability

The raw data supporting the conclusions of this article will be made available by the authors, without undue reservation.
